# Acute Appendicitis Secondary to Intestinal Schistosomiasis

**DOI:** 10.1155/cris/4115372

**Published:** 2025-09-09

**Authors:** Ethan Shyu, Luis Arias-Espinosa, Gabriele Barrocas, Scott Weisenberg, Flavio Malcher

**Affiliations:** ^1^Division of General Surgery, New York University Langone Health, New York, New York, USA; ^2^Division of Infectious Diseases and Immunology, New York University Langone Health, New York, New York, USA; ^3^Division of General Surgery, Lenox Hill Hospital, Northwell, New York, USA

## Abstract

Schistosomiasis is a parasitic disease caused by blood flukes commonly found in sub-Saharan Africa and select other areas in Asia and the Americas. The disease can manifest in a wide range of acute and chronic conditions, rarely presenting as acute appendicitis. Herein we report a case of a 36-year-old female patient from a nonendemic area (New York City) with a history of travel presenting with acute appendicitis secondary to instestinal schistosomiasis.

## 1. Introduction

Appendectomy for acute appendicitis is one of the most common abdominal surgeries performed worldwide [1]. Most cases arise from fecal stasis, fecaliths, and lymphoid hyperplasia; however, appendicitis can rarely be secondary to intestinal parasitic infections [2]. Common parasites include *Entameba histolytica*, *Giardia lamblia*, and *Trichuris trichiura* occurring in 0.061%, 0.034%, and 0.025% of appendectomy samples, respectively [2]. Besides these, blood flukes predominately found in Africa and South Asia can also commonly cause schistosomiasis [3]. Schistosomal-associated appendicitis (SAA) in nonendemic areas is nevertheless rare, with the disease having a prevalence of 0.3% [4]. Patients with SAA commonly present with fever, abdominal pain, or vomiting [5]. SAA represents a surgical emergency, and an appendectomy paired with antihelminthic therapy is crucial for treatment [6]. Herein, we report a 34-year-old female patient in the United States presenting with acute appendicitis later diagnosed as SAA.

## 2. Case Presentation

**Figure 1:**
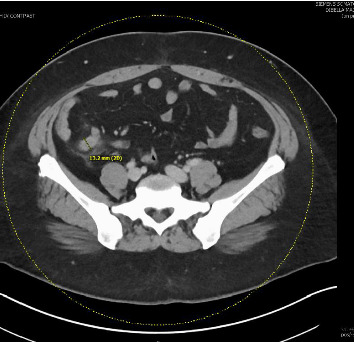
CT scan. Distention of the appendix up to 1.3 cm in caliber with periappendiceal inflammatory fat stranding, suggestive of uncomplicated acute appendicitis. No bowel obstruction.

A 36-year-old female patient presented at the emergency department with chills, a low-grade fever of 37.3°C, and periumbilical abdominal pain lasting for more than a day. Significant past medical history included anemia, complex endometrial hyperplasia with atypia, endometrial polyp, polycystic ovary syndrome, hyperlipidemia, hypertension, and prediabetes. Laboratory results revealed a white blood cell count of 8.6, an absolute eosinophil count of 0.1, and positive urinalysis with a red blood cell count of 3/HPF, an elevated white blood cell count of 50/HPF, and a bacteria count of few/HPF. CT revealed hepatic steatosis with splenomegaly without evidence of portal tract fibrosis and a distension of the appendix up to 1.3 cm in caliber with periappendiceal inflammatory fat stranding suggestive of uncomplicated acute appendicitis (Figure 1). The patient received ceftriaxone and metronidazole and underwent a robotic appendectomy.

The procedure was without complication; however, a nodular lesion was found on the tip of the appendix. Surgical pathology returned with a pseudocyst with calcification suspicious for *Schistosoma* eggs as well as extensively fibrotic, focally hemorrhagic mucosa with fibrous obliteration of the lumen throughout the midportion and distal tip. Furthermore, multiple diverticula were present within the midportion and distal tip, ranging from 0.3 to 1.1 cm in greatest dimension and were all extensively fibrotic. Given these findings, the patient was referred to infectious diseases. *Schistosoma* antibody IgG by ELISA was negative, and stool testing for ova and parasites was also negative. The patient was given praziquantel.

The patient grew up in New York, a nonendemic area, and has continued to live there for her entire life. However, upon reflection, the patient admitted to traveling to Turkey, the Mediterranean, Puerto Rico, and Mali—the last of which we believe is where the patient was first exposed to the disease 20 years ago. Follow-up revealed no postoperative complications, with the patient remaining well.

## 3. Discussion

SAA is clinically indistinguishable from other types of appendicitis. Furthermore, hematological and biochemical tests have limited sensitivity in detecting the disease [7]. Serology and stool ova and parasite testing constitute the typical diagnostic workup for SAA [8]. For surgical specimens, identification of species-specific characteristics of *Schistosoma* eggs may also be helpful [9]. CT features, such as appendicular diameter more than 1.4 cm, appendicular wall calcifications, cecal calcifications, and pneumatosis may be early indicator signs of SAA [10]. In the case of our patient, CT revealed no evidence of appendicular wall calcification, cecal calcification, or pneumatosis; however, appendicular distension measured up to 1.3 cm. Ultimately, diagnosis of SAA depends on histopathologic confirmation of schistosomal ova and granulomatous inflammation in the appendix [6]. Praziquantel is the drug of choice for treatment of schistosomiasis, with a dosage of 40–60 mg/kg depending on species [11].

Schistosomiasis is caused by trematodes of the genus *Schistosoma*. Found in freshwater rivers or lakes, the eggs of these parasites will hatch and release miracidia, which then burrow into select snail species and develop sporocysts. Once the sporocysts are fully grown into cercariae, they will be rereleased into the water wherein they penetrate human skin. Circulation of the schistosomulae occurs via venous circulation to the lungs, heart, and then to the liver. Once fully mature, the schistosumulae will exit the liver to a final location dependent on the species of *Schistosoma*. The two variants responsible for SAA, *S. mansoni* and *S. japonica*, typically reside in the inferior and the superior mesenteric veins, respectively. Once this has occurred, female schistosomulae will lay eggs in the small venules of the portal and perivescial systems, which depending on the location of the adult, will then travel to the lumen of the intestine or the bladder to be excreted in stool or urine, respectively [12].

Some patients will present with acute schistosomiasis, also known as Katayama fever, a systemic hypersensitivity reaction occurring a few weeks after infection; however, most patients will be asymptomatic [12]. For example, a recent study on schistosomiasis in Norwegian students traveling to Africa suggested that only 2%–10% of individuals that acquired the infection present with any symptoms. Importantly, this study also suggests that in nonendemic regions, the infection is more common than is clinically appreciated [13].

Chronic disease in endemic areas is the result of an immune reaction to the eggs and manifests as syndromes such as esophageal variceal bleeding or hematuria [14]. Occasionally egg migration causes other clinical syndromes, as occurred in our patient. However, for most of the disease course, our patient was asymptomatic with no reports of abdominal pain or diarrhea despite likely having the disease for almost 20 years. These findings are consistent with the lab results suggesting a light burden of disease.

The pathophysiological mechanism of SAA is not fully known; however, it is generally believed that there are two mechanisms: granulomatous and obstructive. Granulomatous SAA is believed to occur in the early stage of schistosomiasis, wherein newly deposited eggs trigger an immunological response destroying both the eggs and the surrounding tissue [7, 15]. Obstructive SAA is believed to occur in late-stage infections, wherein chronic inflammation from calcified eggs leads to the obstruction of the appendiceal lumen. This obstruction is then believed to increase the risk of superadded infection, particularly intestinal tuberculosis, ileocecal intussusceptions, and intestinal obstruction [10, 15]. In the case of our patient, obstructive SAA is believed to have been the mechanism at play, as the patient had been asymptomatic with the disease for almost two decades. Furthermore, tissue necrosis and eosinophilia typically associated with a granulomatous reaction were not found during the appendectomy [10].

Given the patient's travel history, it was initially believed that the infection was acquired on visitation to Puerto Rico. Interestingly, *Schistosoma* is not native to the Americas, as the parasitic infection was first introduced to the region by the Trans-Atlantic slave trade [16]. Fortunately, in Puerto Rico and other parts of the Caribbean, public health measures aimed at snail control and improvements in water availability and sanitation have effectively eliminated the disease from the region [17]. As such, we believe it unlikely that our patient acquired the disease in Puerto Rico, with Mali being the more likely candidate.

Approximately 5%–10% of patients with schistosomial infection develop hepatosplenic schistosomiasis, a condition caused by ova trapped in the presinusoidal periportal spaces [18]. The granulomatous reaction progresses to portal hypertension and periportal fibrosis, which may present in patients as moderate splenomegaly and enlargement of the left hepatic lobe [18]. In this case, it is unlikely that the patient's splenomegaly was related to the *Schistosomiasis* given the low burden of infection and the absence of periportal fibrosis on imaging.

Ultimately, this case highlights that schistosomal appendicitis can arise in patients even in nonendemic areas and that clinicians should maintain suspicion of this disease, especially among patients with a history of travel.

## Data Availability

Data sharing is not applicable to this article, as no new data were created or analyzed in this study.
